# Risk and Protective Factor Indicators Management Committee: methodological approach in the construction of indicators of the interagency health information network (Ripsa), 2023

**DOI:** 10.1590/1980-549720260026

**Published:** 2026-07-27

**Authors:** Deborah Carvalho Malta, Max Moura de Oliveira, Mariana Gonçalves de Freitas, Anna Beatriz Souza Antunes, Letícia Mendes Ricardo, Vera Lucia Tierling, Debora França dos Santos, Paula Carvalho de Freitas, Flora Vitória Serena Oliveira Baldi, Bárbara Aguiar Carrato, Ana Lorena Lima Ferreira, Thanise Sabrina Souza Santos, Gabriela Oliveira, Isabela da Costa Gaspar da Silva, Luiz Antônio Alves de Menezes-Júnior, Luci Fabiane Scheffer Moraes, Management Committee on Risk and Protective Factors Indicators

**Affiliations:** I Universidade Federal de Minas Gerais, School of Nursing, Department of Maternal-Child Nursing and Public Health – Belo Horizonte (MG), Brazil.; II Universidade Federal de Goiás, Instituto de Patologia Tropical e Saúde Pública, Department of Collective Health – Goiás (GO), Brazil.; III Ministry of Health, Secretariat of Health Surveillance and Environment – Brasília (DF), Brazil.; IV Universidade Federal de Minas Gerais, School of Nursing – Belo Horizonte (MG), Brazil.; V Ministry of Health, Secretariat of Primary Health Care – Brasília (DF), Brazil.; VI Ministry of Health, Secretariat of Information and Digital Health – Brasília (DF), Brazil.

**Keywords:** Health status indicators, Risk factors, Protective factors, Health surveys, Health Information Systems, Brazil

## Abstract

**Objective:**

To describe the role of the Risk and Protective Factors Indicator Management Committee (RPF CGI) in the resumption and update of the Interagency Health Information Network (*Rede Interagencial de Informações para a Saúde* – Ripsa) indicator matrix in 2023, highlighting the sustainability of data sources and validation processes.

**Methods and Results:**

Interinstitutional technical meetings were held between 2023 and 2025, involving 36 institutions, to review, define, and validate the indicators using a standardized indicator qualification form. A consensus was reached to use data from the Surveillance System for Risk and Protective Factors for Chronic Diseases by Telephone Survey (*Sistema de Vigilância de Fatores de Risco e Proteção para Doenças Crônicas por Inquérito Telefônico* – Vigitel), National Study of Infant Feeding and Nutrition (*Estudo Nacional de Alimentação e Nutrição Infantil –* Enani), Live Birth Information System (*Sistema de Informações sobre Nascidos Vivos –* Sinasc), National Health Survey (*Pesquisa Nacional de Saúde –* PNS), Household Budget Survey (*Pesquisa de Orçamentos Familiares –* POF), National School Health Survey (*Pesquisa Nacional de Saúde do Escolar –* PeNSE), National Demographic and Health Survey (*Pesquisa Nacional de Demografia e Saúde –* PNDS), Annual Industrial Survey – Product (*Pesquisa Industrial Anual – Produto –* PIA-Produto), National Survey on Health and Nutrition (*Pesquisa Nacional sobre Saúde e Nutrição –* PNSN), and National Oral Health Survey (*Pesquisa Nacional de Saúde Bucal –* SB Brasil). The matrix was expanded to 39 indicators, distributed across nine dimensions: noncommunicable chronic diseases, alcohol consumption, smoking, physical activity, nutritional status, dietary intake, childbirth, violence, and oral health.

**Conclusion:**

The RPF CGI contributed to the qualification and sustainability of Ripsa indicators, which are essential for monitoring public policies, supporting evidence-based decision-making, and addressing health inequalities, aligning with national and international agendas such as the Sustainable Development Goals.

## INTRODUCTION

The Interagency Health Information Network (*Rede Interagencial de Informações para a Saúde* – Ripsa) is a collaborative initiative established in 1996 to produce, validate, and disseminate strategic public health information in Brazil. It was created through a partnership involving the Ministry of Health (MoH), the Pan American Health Organization/World Health Organization (PAHO/WHO), the National Council of Health Secretaries (*Conselho Nacional de Secretários de Saúde* – Conass), and the National Council of Municipal Health Secretariats (*Conselho Nacional de Secretarias Municipais de Saúde* – Conasems), in collaboration with academic institutions, research centers, and governmental agencies. Ripsa has become a key forum for the generation of information to support the formulation, management, and evaluation of policies and actions within the Brazilian Unified Health System (*Sistema Único de Saúde* – SUS)^1^.

After nearly two decades of continuous operation, the Network entered a period of inactivity beginning in 2015. This hiatus persisted until 2023, when activities were resumed under the Secretariat of Information and Digital Health (*Secretaria de Informação e Saúde Digital* – Seidigi/MoH), which became responsible for its development and coordination. The official resumption occurred in August 2023, reaffirming Ripsa’s role as a qualified technical body for health information^2^.

Ripsa currently comprises 45 governmental and non-governmental institutions, with the voluntary participation of hundreds of specialized professionals. This structure ensures methodological rigor and technical legitimacy in the consensus-building process and the periodic updating of a set of 184 Basic Health Indicators (BHI). The Indicator Management Committees (*Comitês de Gestão de Indicadores* – CGI) are permanent bodies responsible for the development and regular review of these indicators, which are organized into seven categories: demographic, socioeconomic, morbidity, mortality, resources, coverage, and risk and protective factors. Each category is coordinated by a technical committee responsible for defining, producing, and analyzing the health indicators^3^.

The participating institutions operate through consensus in the production, analysis, and dissemination of data, information, and indicators related to health conditions and their determinants, thereby promoting knowledge of the Brazilian health situation and supporting monitoring and decision-making within SUS^1^. The CGI on Risk and Protective Factors (RPF) was created in 2011 during the 21^st^ Interagency Workshop (*Oficina de Trabalho Interagencial* – OTI), the highest decision-making body for technical definitions within Ripsa. The establishment of this CGI was essential to expand the monitoring of RPF indicators^4^. Initially, Ripsa indicators were organized according to the classification proposed in the document *Situación de Salud en las Américas: Indicadores Básicos 1995*, including the groups of mortality, morbidity, and risk factors^5^. Subsequently, morbidity and risk factor indicators were separated. In August 2023, the RPF CGI was reestablished, with the participation of 36 teaching and research institutions, coordinated by Universidade Federal de Minas Gerais (UFMG), with reporting by the Department of Epidemiological Analysis and Surveillance of Noncommunicable Diseases (*Departamento de Análise Epidemiológica e Vigilância de Doenças Não Transmissíveis* – Daent) of the Secretariat of Health and Environmental Surveillance (SVSA) of the Ministry of Health of Brazil^6^.

Given this context, the objective was to describe the role of the Risk and Protection Factor Indicator Management Committee in the resumption of Ripsa in 2023, as well as to present its contributions and the composition of the indicators under its responsibility. The article also details the methodological processes of agreement, validation, and sustainability of the data sources that underpin Ripsa’s matrix of risk and protection factor indicators.

## Methods AND RESULTS

Six meetings of the RPF CGI were held between 2023 and 2025, involving representatives from 36 governmental and non-governmental institutions. The objectives included reviewing, selecting, and validating indicators; defining data sources and calculation methods; testing and assessing monitoring feasibility; preparing indicator specification sheets; and reaching consensus on the indicator matrix. During these meetings, each indicator was systematically evaluated for relevance, sustainability, and feasibility, taking into account methodological and operational specificities.

The standardized indicator qualification form from Ripsa was adopted. This form includes mandatory fields such as name, concept, justification, calculation method, analysis categories, periodicity, and data source, thereby ensuring transparency, standardization, and reproducibility of information. The instrument was developed collectively based on consensus among participating institutions, with the aim of ensuring comparability and quality of the information produced.

In the process of developing and reviewing the indicators, all stages complied with the ethical principles established in current Brazilian legislation. Only secondary, publicly available, de-identified data were used, with no possibility of individual participant identification. Consequently, submission to the Research Ethics Committees (*Comitês de Ética em Pesquisa* – CEP/Conep) system was not required, in accordance with Resolution No. 466, of December 12, 2012, of the National Health Council^7^.

The criteria for selecting and validating the indicators included relevance to public health, sustainability of the data source, operational feasibility, potential for temporal and territorial monitoring, and alignment with national and international agendas.

The indicators of RPF CGI were constructed using data extracted from various national surveys and health information systems, encompassing distinct populations, methodologies, and data collection periods. The primary data sources included:

a) Surveillance System for Risk and Protective Factors for Chronic Diseases by Telephone Survey (*Sistema de Vigilância de Fatores de Risco e Proteção para Doenças Crônicas por Inquérito Telefônico* – Vigitel): conducted between 2006 and 2023. Vigitel is a population-based telephone survey, conducted annually by the Ministry of Health, collecting information on noncommunicable chronic diseases (NCDs) and their main RPF in adults aged 18 and over in Brazilian capitals. In the editions carried out between 2006 and 2019, a minimum sample size of approximately 2,000 individuals was established in each city, via landline telephone. However, in 2020 and 2021, due to difficulties imposed by the COVID-19 pandemic on data collection, a reduced sample size of approximately 1,000 individuals in each city was established. For 2023, 800 interviews were conducted in each of the locations, and mobile phone interviews were included in the sample due to the decline in landline telephone coverage. Post-stratification weights by gender, age, and education are used to represent adults in capital cities^8^.
b) The National Health Survey (*Pesquisa Nacional de Saúde –* PNS) is the most comprehensive health survey conducted in Brazil, with editions carried out in 2013 and 2019. It is based on a subsample of the Master Sample of the Integrated Household Survey System (*Sistema Integrado de Pesquisas Domiciliares –* SIPD) of the Brazilian Institute of Geography and Statistics (*Instituto Brasileiro de Geografia e Estatística* – IBGE), using three-stage cluster sampling (primary sampling units, households, and individuals). The survey was designed to collect information across the following domains: health conditions; health care (including access, utilization, continuity of care, and financing); surveillance of NCDs and associated RF; as well as issues related to social inequalities in health^9^.
c) The Household Budget Survey (*Pesquisa de Orçamentos Familiares –* POF), conducted by IBGE since 1961, has included information on food purchases since the 2002/2003 edition, as well as data on individual food consumption in the 2008/2009 and 2017/2018 editions. POF examines household budgets in conjunction with additional information on the living conditions of Brazilian families, price indices, and consumption-based living standards. It also encompasses studies on individual food consumption and analyses of household food availability^10^.
d) The National School Health Survey (*Pesquisa Nacional de Saúde do Escolar* – PeNSE), conducted in 2009, 2012, 2015, and 2019, is the most important survey targeting the adolescent population in Brazil. From 2015 onward, PeNSE began to represent schoolchildren aged 13 to 17 years through a subsample, and, in 2019, this age group comprised approximately 150,000 students from public and private schools across the country. PeNSE aims to determine the prevalence of behavioral risk factors among adolescents; monitor temporal trends in these prevalences; and generate evidence to inform and evaluate interventions^11,12^.
e) The National Demographic and Health Survey (*Pesquisa Nacional de Demografia e Saúde* – PNDS), conducted in 1996 and 2006, characterizes the profile of women of reproductive age and children under five years of age in Brazil, as well as identifies changes in the health and nutritional status of these populations. Data were collected through household interviews, including anthropometric measurements (height, weight, and waist circumference), blood sample collection, and information on the use of iodized salt. In 2006, the sample comprised 15,000 women and approximately 5,000 children under 5 years of age, representing the 5 Brazilian macro-regions and both urban and rural settings^13^.
f) The National Food and Nutrition Survey (*Estudo Nacional de Alimentação e Nutrição Infantil* – Enani) is a population-based household survey, with its first edition conducted in 2019 using a probabilistic sample of children under 5 years of age. The sample was distributed across 123 municipalities in the 26 Federative Units and the Federal District and included 14,558 children under 5 years of age. Enani encompasses indicators of child nutrition and health, including breastfeeding practices and nutritional status^14^.
g) The Annual Industrial Product Survey (*Pesquisa Industrial Anual – Produto* – PIA-Produto) is part of the Economic Statistics Modernization Program of IBGE and aims to meet the growing demand for statistical information on industrial goods and services produced in the country. Initiated in 1998, the PIA-Produto provides updated data on the production of industrial goods and services, in terms of both quantity and value, for products manufactured by the country’s main companies. The data analyzed refer to the production, importation, and consumption of alcohol in Brazil^15^.
h) The National Health and Nutrition Survey (*Pesquisa Nacional de Saúde e Nutrição* – PNSN), conducted in 1989, was based on a nationally representative household sample. PNSN enabled the collection of information on breastfeeding, nutritional status, and household characteristics, among other variables, thereby contributing to the assessment of food and nutrition conditions and to the planning of public policies^16–18^.
i) The National Oral Health Survey (*Pesquisa Nacional de Saúde Bucal* – SB Brasil) is a nationwide epidemiological survey of oral health conducted by the Ministry of Health. Using probabilistic cluster sampling, it is representative of major regions, Federative Units, and state capitals, and encompasses multiple age groups. The survey allows comparability between the 2010 and 2023 editions. Based on interviews and clinical examinations, indicators such as dental caries, clinical consequences of untreated caries (PUFA), and prosthetic needs, among others, were assessed^19^.
j) The Live Birth Information System (*Sistema de Informações sobre Nascidos Vivos* – Sinasc) compiles data derived from the Live Birth Certificate, including information on the type of delivery, gestational characteristics, and maternal and neonatal attributes, among others. These data are essential for the construction of indicators such as the infant mortality rate, early and late neonatal mortality, frequencies related to newborn risk, number of prenatal consultations, and fertility rates by place of occurrence. Such information enables the estimation of specific demands for the organization of health services, including the need for neonatal intensive care^16^. Sinasc was officially implemented in 1990 and consolidates data on births registered throughout the national territory, providing information on birth rates at all levels of SUS^17^.

**Figure 1:**
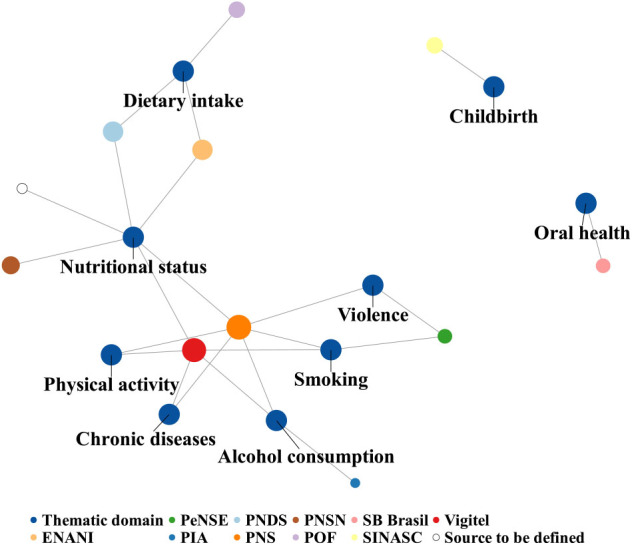
Network of connections among nine thematic domains and data sources for indicators of risk and protective factors, Ripsa, Brazil, 2025.

The indicator matrix of RPF CGI has undergone expansion and restructuring over time. Until 2011, it comprised 17 indicators. In the most recent revision, 8 new indicators were proposed, and, as determined by the 31^st^ OTI, each distinct data source is now considered a separate indicator. By 2025, the matrix will comprise 39 risk and protective factor indicators, organized into 9 thematic dimensions. Figure 1 illustrates the relationships between these dimensions and the indicators’ data sources, highlighting that the size of each source node is directly proportional to the number of indicators that use it as a reference. In other words, the greater the number of indicators associated with a given data source, the larger its node in the graphical representation. The figure underscores the relevance of PNS and Vigitel as primary data sources for multiple indicators, accounting for 11 and 10 indicators, respectively.

The network analysis highlights the central role of these two sources in ensuring the sustainability of the monitoring system and in supporting a balanced distribution of indicators across the nine dimensions.

Details regarding the code, name, concept, data sources, and analytical variables for each indicator are presented in Chart 1 and, in greater detail, in Supplementary Material 1. Complete qualification sheets are available on the Ripsa website^20^. The disaggregation of indicators varies according to the data source but generally includes geographic unit (regions, states, Federal District, and state capitals), age, gender, and race/skin color. When available, additional stratification by education and income is also included, serving as proxies for socioeconomic status.

**Chart 1:** Matrix of indicators of risk and protective factors across nine thematic domains, Ripsa, Brazil, 2025.

Code and name	Concept	Data sourcesdados	Categories of analysis
RPF.1.01.1: Prevalence of self-reported diabetes mellitus in individuals aged 18 years old or older.	Prevalence of diabetes mellitus based on self-reported medical diagnosis in individuals aged 18 years old or older.	Vigitel 2006 to 2023	**Vigitel categories “**
RPF.1.01.2: Prevalence of self-reported diabetes mellitus in individuals aged 18 years old or older.	Prevalence of diabetes mellitus based on self-reported medical diagnosis in individuals aged 18 years old or older.	PNS 2013, 2019	**PNS categories“”**
RPF. 1.02.1: Prevalence of self-reported arterial hypertension in individuals aged 18 years old or older.	Prevalence of self-reported arterial hypertension in individuals aged 18 years old or older.	Vigitel 2006 to 2023	**Vigitel categories“**
RPF.1.02.2: Prevalence of self-reported arterial hypertension in individuals aged 18 years old or older.	Prevalence of arterial hypertension based on self-reported medical diagnosis in individuals aged 18 years old or older.	PNS 2013, 2019	**PNS categories“”**
RPF.2.01.1: Prevalence of individuals aged 18 years old or older reporting heavy episodic alcohol consumption.	Percentage of individuals aged 18 years old or older reporting heavy episodic alcohol consumption in the last 30 days.	Vigitel 2006 to 2023	**Vigitel categories“**
RPF.2.01.2: Prevalence of individuals aged 18 years old or older reporting heavy episodic alcohol consumption.	Percentage of individuals aged 18 years old or older reporting heavy episodic alcohol consumption in the last 30 days.	PNS 2013, 2019	**PNS categories“”**
RPF.2.02.1: Prevalence of individuals aged 18 years old or older driving motor vehicles after alcohol consumption.	Percentage of individuals aged 18 years old or older reporting driving after consuming any amount of alcohol.	Vigitel 2006 to 2023	**Vigitel categories“**
RPF.2.02.2: Prevalence of individuals aged 18 years old or older driving motor vehicles after alcohol consumption.	Percentage of car or motorcycle drivers who drove immediately after alcohol consumption in the last 12 months.	PNS 2013, 2019	**PNS categories“”**
RPF.2.03: Alcohol consumption in liters of pure alcohol per capita (15 years or more) per year.	Annual per capita consumption of pure alcohol (15 years or more).	PIA-Produto, COMEX STAT and UN Tourism	Not applicable
RPF.3.01.1: Prevalence of current smokers aged 18 years old or older.	Prevalence of individuals aged 18 years old or older who reported smoking.	Vigitel 2006 to 2023	**Vigitel categories“**
RPF.3.01.2: Prevalence of individuals aged 18 years old or older who reported smoking.	Prevalence of individuals aged 18 years old or older who reported smoking.	PNS 2013, 2019	**PNS categories“”**
RPF.3.02.1: Prevalence of current use of ESD# in individuals aged 18 years old or older.	Prevalence of current use of ESD# among individuals aged 18 years old or older.	Vigitel 2019 to 2023	**Vigitel categories“**
RPF.3.02.2: Prevalence of current use of ESD# in individuals aged 18 years old or older.	Prevalence of current use of ESD# among individuals aged 18 years old or older.	PNS 2013, 2019	**PNS categories“”**
RPF.3.02.3 Prevalence of current use of ESD# among students aged 13 to 17 years old.	Prevalence of students aged 13 to 17 years old reporting ESD# use in the last 30 days.	PeNSE	Gender; Age range: 13 to 15, 16 to 17 years); Race/skin color: White, Black, Brown
RPF.3.03.1: Prevalence of former smokers aged 18 years old or older.	Prevalence of individuals aged 18 years old or older who are former smokers.	Vigitel 2006 to 2023	**Vigitel categories“**
RPF.3.03.2: Prevalence of former smokers aged 18 years old or older.	Prevalence of individuals aged 18 years old or older who are former smokers.	PNS 2013, 2019	**PNS categories“”**
RPF.4.01.1: Prevalence of recommended physical activity during leisure time in individuals aged 18 years old or older.	Prevalence of individuals aged 18 years old or older meeting recommended levels of leisure-time physical activity.	Vigitel 2006 to 2023	**Vigitel categories“**
RPF.4.01.2: Prevalence of physical activity during leisure time in individuals aged 18 years old or older.	Prevalence of individuals aged 18 years old or older meeting recommended levels of leisure-time physical activity.	PNS 2013, 2019	**PNS categories“”**
RPF.4.02.1: Prevalence of insufficient physical activity in individuals aged 18 years old or older.	Prevalence of insufficient physical activity in individuals aged 18 years old or older.	Vigitel	**Vigitel categories“**
RPF.4.02.2: Prevalence of insufficient physical activity in individuals aged 18 years old or older.	Prevalence of insufficiently active individuals aged 18 years old or older.	PNS 2013, 2019	**PNS categories“”**
RPF.5.01.1: Prevalence of overweight in individuals aged 18 years old or older.	Prevalence of overweight in individuals aged 18 years old or older.	Vigitel 2006 to 2023 (except 2022)	**Vigitel categories“**
RPF.5.01.2: Prevalence of overweight in individuals aged 18 years old or older.	Prevalence of overweight in individuals aged 18 years old or older.	PNS 2013, 2019	**PNS categories“”**
RPF.5.02: Prevalence of overweight in adolescents.	Not available for indicator development.		
RPF.5.03: Prevalence of overweight in children under 5 years of age.	Percentage of children <5 years of age with overweight.	PNSN 1989, PNDS 1996 and 2006, Enani 2019	Gender; National economic indicator; Mother’s race/skin color: White, Black, Brown, Yellow and Indigenous
RPF.5.04: Prevalence of underweight-for-age in children under 5 years of age.	Percentage of children <5 years of age with underweight-for-age. Underweight = values below a z-score of -2 or below the 3^rd^ percentile of the weight-for-age (W/A) index, according to WHO growth curves.	PNSN 1989, PNDS 1996 and 2006, Enani 2019	Gender; National economic indicator; Mother’s race/skin color: White, Black, Brown, Yellow and Indigenous
RPF.5.05: Prevalence of stunting in children under 5 years of age.	Percentage of resident children <5 years of age with stunting-for-age. Stunting = values below the 3^rd^ percentile or a z-score of -2 of the height-for-age (H/A) index, according to WHO growth curves.	PNSN 1989, PNDS 1996 and 2006, Enani 2019	Gender; National economic indicator; Mother’s race/skin color: White, Black, Brown, Yellow and Indigenous
RPF.5.06: Prevalence of double burden of malnutrition in children under 5 years of age.	Percentage of children <5 years of age with the double burden of malnutrition, defined by BMI-for-age classified as severe thinness/thinness or overweight/obesity, according to WHO child growth standards.	PNSN 1989, PNDS 1996 and 2006, Enani 2019	Gender; National economic indicator; Mother’s race/skin color: White, Black, Brown, Yellow and Indigenous
RPF.6.01: Daily per capita relative share of calories from fruits, vegetables, and legumes in the total calories of foods acquired in the household.	Daily per capita relative share of calories from FVL in the total calories of foods acquired in the household for consumption by the resident population.	POF 2008-2009 and 2017-2018	Gender; National Economic Indicator quintile: 1^st^, 2^nd^, 3^rd^, 4^th^, and 5^th^ quintile; Race/skin color: White, Black, Brown, Yellow, and Indigenous
RPF.6.02: Relative share of fruits, vegetables, and legumes in total caloric intake based on individual dietary consumption. ^@^	Daily per capita relative share of calories from fruits, vegetables, and legumes in total caloric intake based on individual dietary consumption.	POF 2008-2009 and 2017-2018	Gender; Household setting: rural and urban; Race/skin color: White, Black, Brown, Yellow, and Indigenous; National economic indicator
RPF.6.03: Relative share of ultra-processed foods in total caloric intake based on individual dietary consumption. ^@^	Daily per capita relative share of calories from ultra-processed foods in total caloric intake based on individual dietary consumption.	POF 2008-2009 and 2017-2018	Gender; Household setting: rural and urban; Race/skin color: White, Black, Brown, Yellow, and Indigenous; National economic indicator
RPF.6.04: Prevalence of exclusive breastfeeding during the first six months of life.	Percentage of resident children aged 0 to 5 months and 29 days (0 to 179 days) who were exclusively breastfed.	PNDS 2006 and Enani 2019	Gender; National economic indicator; Mother’s race/skin color: White, Black, Brown, Yellow, and Indigenous; Household setting: urban and rural.
RPF.6.05: Prevalence of continued breastfeeding in children under 2 years of age.	Percentage of resident children <2 years of age who received breast milk, directly from the breast or expressed, alongside complementary feeding.	PNDS 2006 and Enani 2019	Gender; National economic indicator; Mother’s race/skin color: White, Black, Brown, Yellow, and Indigenous; Household setting: urban and rural.
RPF.7.01: Proportion of live births by maternal age.	Presents the proportion of live births by maternal age in relation to the total number of live births.	Sinasc	Geographic unit: Brazil, major regions, states, Federal District, and capital cities; Maternal age ranges up to 19, 20-34, 35-39, 40+ years
RPF.7.02: Proportion of live births with low birth weight by gestational age at delivery.	Proportion of live births weighing <2,500 grams, according to gestational age at the time of delivery.	Sinasc	Gestational age: preterm, term/post-term, or post-term.
RPF.7.03: Prevalence of live births with congenital anomalies.	Number of live births with mention of at least one congenital anomaly relative to the total number of registered live births.	Sinasc	Geographic unit: Brazil, major regions, states, Federal District, and capital cities.
RPF.8.01: Prevalence of violence against women aged 18 years old or older.	Percentage of women ≥18 years who reported having experienced at least one type of violence investigated in the past 12 months.	PNS 2013, 2019	**PNS categories“”**
RPF.8.02: Prevalence of sexual violence against girls aged 13 to 17 years.	Prevalence of girls aged 13 to 17 years who reported having experienced sexual violence at any point in their lives.	PeNSE	Age range: 13 to 15 years and 16 to 17 years; Race/skin color: White, Black, and Brown.
RPF.9.01: DMFT Index (number of decayed, missing, and filled teeth) at 12 years of age.	Mean number of permanent teeth that are decayed, missing (extracted), and filled (restored) at 12 years of age.	SB Brasil	Year: 1996, 2003, 2010, and 2023.
RPF.9.02: Proportion of children aged 5 to 6 years who are caries-free (dmft index = 0)	Percentage of children aged 5 to 6 years with a dmft index = 0.	SB Brasil	Year: 1996, 2003, 2010, and 2023.

BMI: Body Mass Index; FVL: fruits, vegetables, and legumes; Vigitel: Surveillance System of Risk and Protective Factors for Chronic Diseases by Telephone Survey (*Sistema de Vigilância de Fatores de Risco e Proteção para Doenças Crônicas por Inquérito Telefônico*); PNS: National Health Survey (*Pesquisa Nacional de Saúde*); PIA-Produto: Annual Industrial Survey – Product (*Pesquisa Industrial Anual Produto*) of the Brazilian Institute of Geography and Statistics (*Instituto Brasileiro de Geografia e Estatística*); COMEX STAT: Brazilian Foreign Trade Data Query and Extraction System of the Ministry of Development, Industry, Trade, and Services (*Sistema de Consultas e Extração de Dados do Comércio Exterior Brasileiro do Ministério do Desenvolvimento, Indústria, Comércio e Serviços*); UN Tourism: tourism statistics of the World Tourism Organization; PeNSE: National School Health Survey (*Pesquisa Nacional de Saúde do Escolar*); PNSN: National Survey on Health and Nutrition (*Pesquisa Nacional sobre Saúde e Nutrição*); PNDS: National Demographic and Health Survey (*Pesquisa Nacional de Demografia e Saúde*); Enani: National Study of Infant Feeding and Nutrition (*Estudo Nacional de Alimentação e Nutrição Infantil*); POF: Household Budget Survey (*Pesquisa de Orçamentos Familiares*); Sinasc: Live Birth Information System (*Sistema de Informações sobre Nascidos Vivos*); SB Brasil: National Oral Health Survey (*Pesquisa Nacional de Saúde Bucal*); #Electronic Smoking Device; “Gender; Age range; Educational level; “”Gender; Age range; Educational level; Household income; Race/skin color; Household setting; Income); @ – Indicator under development.

### Data availability statement

The entire dataset supporting the results of this study has been published in the article and in the “Supplementary Materials” section.

## Discussion

The study examined the relevance of the methodological process conducted by RPF CGI in the resumption of Ripsa, culminating in an indicator matrix aligned with contemporary challenges in Brazilian public health. The reactivation of the Network in 2023 followed a hiatus of nearly a decade, a period marked by significant political instability and the implementation of fiscal austerity measures, such as Constitutional Amendment No. 95, which imposed substantial constraints on the financing of a universal health system that, despite its historical advances, continues to face persistent challenges^21,22^.

In this adverse context, the articulatory capacity of the public health community proved resilient. An illustrative example is the development of the “Special Notebook of Basic Indicators for Monitoring COVID-19,” an initiative that applied the Network’s collaborative approach and technical expertise to address the health emergency, even in the absence of formal endorsement by Ripsa^23^. Thus, the official resumption of the Network represents not merely a bureaucratic reactivation but also a political reaffirmation of the importance of evidence-based governance for the strengthening and sustainability of SUS.

The approach adopted by the committee reflects a high degree of flexibility in aligning national surveillance with both global and local priorities, thereby ensuring the relevance of the information generated. The indicator matrix is directly aligned with the targets established in the Sustainable Development Goals (SDGs)^24,25^, the WHO’s Global Action Plan for the Prevention and Control of NCDs^26^, and, fundamentally, the Strategic Action Plan for Coping with NCDs in Brazil 2011-2022^26^ and the Strategic Action Plan for Coping with NCDs in Brazil 2021-2030^26^. This capacity to review and adapt indicators is essential for ensuring that data effectively support Primary Health Care (PHC), which serves as the coordinator of care and the primary locus for implementing prevention and control actions for these conditions. The availability of consistent and regularly produced data strengthens territorial planning and the evaluation of programs within PHC and health promotion strategies^26,27^.

The design of the matrix prioritizes data sources of recognized quality and methodological robustness, constituting a fundamental pillar for the credibility of the indicators. Brazil’s health survey system, given its complexity and scope, represents a strategic asset for surveillance, and the centrality of PNS and Vigitel, as evidenced by the network analysis, confirms their complementary and synergistic roles. PNS provides depth and national representativeness, being essential for analyses of social, racial, and regional health inequalities^28,29^. In turn, Vigitel, whose validity and accuracy have been consistently demonstrated in comparative studies^30,31^, ensures timeliness and broad coverage for the annual monitoring of trends in state capitals, representing an agile and cost-efficient surveillance tool. The sustainability of these data sources is therefore critical. However, important challenges remain, including the absence of regular nutritional monitoring for adolescents — a critical gap that limits surveillance of risk factors during a pivotal stage of life^32^ — as well as challenges inherent to maintaining high participation rates in surveys in a context of evolving communication patterns^33^.

The organization of the matrix into thematic dimensions constitutes an additional methodological strength. This structure segments data complexity in a coherent manner, aligning with international best practices for organizing health information systems, which advocate logical frameworks to facilitate the analysis and use of information^34^. The indicators related to NCDs, smoking, alcohol consumption, inadequate diet, and physical activity enable integrated monitoring of the principal modifiable risk factors. Brazil is regarded as a success case in tobacco control, reflecting the implementation of robust regulatory policies^35^; however, it continues to face challenges related to interference from the tobacco industry, which seeks, for instance, to introduce new products such as electronic smoking devices^35^. In contrast, alcohol consumption remains elevated, and physical inactivity constitutes a global public health concern^36^, underscoring the need for continuous surveillance to support health promotion policies and the development of healthy environments.

The dimensions of Nutritional Status and Food Consumption reflect the country’s complex nutritional transition, characterized by the coexistence of undernutrition and overweight, known as the double burden of malnutrition^37^. Monitoring the consumption of both healthy and unhealthy foods is essential for assessing the impact of an obesogenic food environment and for informing regulatory policies, such as food taxation and labeling^38,39^.

The Labor and Birth dimension, based on the robust Sinasc, is essential for monitoring maternal and child health. It enables the assessment of aspects ranging from the quality of prenatal care to outcomes with lifelong implications, such as low birth weight^40,41^.

The inclusion of the Violence and Oral Health dimensions represents a significant advancement, expanding the scope of surveillance beyond traditional risk factors. Violence, particularly against women, constitutes a major public health problem with deep social determinants, and its measurement through surveys is essential to ensure visibility of the phenomenon and to support intersectoral protection policies^42^. The indicators included in the matrix are closely aligned with SDG 5 (target 5.2), which aims to eliminate all forms of violence against women and girls^43^. Oral health, in turn, serves as a sensitive marker of social inequalities, and the monitoring of indicators such as the prevalence of dental caries enables the assessment of access to services and the impact of preventive policies^44^.

This multidimensional organization not only facilitates data interpretation but also optimizes the engagement of subject-matter experts, thereby promoting deeper technical understanding within each domain.

When using multiple data sources, it is essential to recognize and address the potential for bias and variation in prevalence estimates arising from differences in sampling strategies and data collection methods. Data from population-based surveys (PNS, Vigitel, PeNSE, Enani, and SB Brasil) are subject to information biases, including recall bias and social desirability bias. These may result in underestimation of risk behaviors, such as low consumption of healthy foods^45^, and overestimation of healthy practices^28,46^, although they do not compromise trend monitoring due to their relative stability over time. Conversely, administrative information systems, such as Sinasc, despite substantial advances in coverage and data quality, may still present inconsistencies in data recording, thereby requiring continuous data quality assurance processes^41^. Transparency in documenting these limitations, detailed in the qualification sheets for each indicator, reflects the commitment of Ripsa to the careful and informed use of information, enabling managers and researchers to appropriately interpret the strengths and limitations of each metric.

Despite inherent limitations, the chosen sources are constantly being improved, reflecting the maturation of health surveillance in Brazil. An emblematic example is Sinasc, which has progressively improved the registration of congenital anomalies, expanding the country’s capacity to monitor these events and plan health care for affected children, including from the perspective of social determinants^47,48^. Similarly, surveys such as PeNSE evolve with each edition to include emerging themes, such as mental health and bullying, demonstrating the adaptability of the surveillance system to respond to new challenges^49^. This continuous effort to improve the quality of the sources reinforces the reliability of the indicator matrix and its usefulness for public health.

The reactivation of Ripsa and the reorganization of RPF CGI represent a critical advancement for Brazilian public health, directly addressing the objective of this article, which is to describe this process. The indicator matrix, supported by high-quality data sources and a collaborative and transparent methodology, is reaffirmed as a strategic instrument for monitoring population health, informing evidence-based policies, and strengthening social accountability. Ensuring its financial and institutional sustainability, as well as the continuous improvement of data sources, is essential for health information to effectively guide action and promote the universal right to health in Brazil.

## SUPPLEMENTARY MATERIAL

The database is not yet available for public use.

## References

[1] Rede Interagencial de Informações para a Saúde (Ripsa) Sobre a Ripsa [Internet].

[2] Rede Interagencial de Informações para a Saúde (Ripsa) Linha do tempo [Internet].

[3] Rede Interagencial de Informações para a Saúde (Ripsa) Comitês de Gestão de Indicadores [Internet].

[4] Brasil. Ministério da Saúde (2011). 23a Oficina de Trabalho Interagencial da Rede Interagencial de Informações para a Saúde: Relatório Técnico. 2011.

[5] Brasil. Ministério da Saúde (1996). Relatório Técnico da Primeira Oficina de Trabalho Interagencial.

[6] Rede Interagencial de Informações para a Saúde (Ripsa) (2023). Relatório Técnico da 29a Oficina de Trabalho Interagencial [Internet].

[7] Conselho Nacional de Saúde (2013). Resolução n° 466, de 12 de dezembro de 2012. Diário Oficial da União.

[8] Brasil. Ministério da Saúde (2024). Vigitel Brasil 2023: vigilância de fatores de risco e proteção para doenças crônicas por inquérito telefônico.

[9] Instituto Brasileiro de Geografia e Estatística (IBGE) (2020). PNS – Pesquisa Nacional de Saúde: 2019 [Internet].

[10] Instituto Brasileiro de Geografia e Estatística (IBGE) (2020). POF – Pesquisa de Orçamentos Familiares: 2017-2018: análise do consumo alimentar pessoal no Brasil [Internet].

[11] Instituto Brasileiro de Geografia e Estatística (IBGE) (2022). PeNSE – Pesquisa Nacional de Saúde do Escolar: análise de indicadores comparáveis dos escolares do 9° ano do ensino fundamental: municípios das capitais: 2009/2019 [Internet].

[12] Malta DC, Morais EAH, Silva AG, Souza JB, Gomes CS, Santos FM (2024). Mudanças no uso do tabaco entre adolescentes brasileiros e fatores associados: Pesquisa Nacional de Saúde do Escolar. Cien Saude Colet.

[13] Brasil. Ministério da Saúde (2009). Pesquisa Nacional de Demografia e Saúde da Criança e da Mulher – PNDS 2006: dimensões do processo reprodutivo e da saúde da criança.

[14] Universidade Federal do Rio de Janeiro (2021). Estudo Nacional de Alimentação e Nutrição Infantil – ENANI-2019: resultados preliminares [Internet].

[15] Instituto Brasileiro de Geografia e Estatística (IBGE) (2023). PIA-Empresa: Pesquisa Industrial Anual – Empresa – Produto: 2021 [Internet].

[16] Brasil. Ministério da Saúde (2025). Sistema de Informações sobre Nascidos Vivos – Sinasc [Internet].

[17] Jorge MHPM, Laurenti R, Gotlieb SLD (2007). Análise da qualidade das estatísticas vitais brasileiras: a experiência de implantação do SIM e do SINASC. Cien Saude Colet.

[18] Instituto Brasileiro de Geografia e Estatística (IBGE) (1990). Pesquisa Nacional sobre Saúde e Nutrição – PNSN, 1989 [Internet].

[19] Brasil. Ministério da Saúde (2023). Pesquisa Nacional de Saúde Bucal: SB Brasil [Internet].

[20] Rede Interagencial de Informações para a Saúde (Ripsa) Lista de indicadores [Internet].

[21] Paim J, Travassos C, Almeida C, Bahia L, Macinko J (2011). The Brazilian health system: history, advances, and challenges. Lancet.

[22] Massuda A, Andrade MV, Martinelli N (2022). Fiscal austerity and the Brazilian health system. Lancet Reg Health Am.

[23] Brasil. Ministério da Saúde (2020). Caderno Especial de Indicadores Básicos para o Monitoramento da Covid-19.

[24] Nações Unidas Brasil (2024). Os Objetivos de Desenvolvimento Sustentável [Internet].

[25] World Health Organization (WHO) (2023). Implementation roadmap 2023–2030 for the WHO global action plan for the prevention and control of noncommunicable diseases 2013–2030.

[26] Brasil. Ministério da Saúde (2021). Plano de Ações Estratégicas para o Enfrentamento das Doenças Crônicas e Agravos Não Transmissíveis no Brasil 2021-2030.

[27] Hone T, Rasella D, Barreto ML, Majeed A, Millett C (2017). The impact of the expansion of primary healthcare on life expectancy and mortality in Brazil: A quasi-experimental study. PLoS Med.

[28] Szwarcwald CL, Malta DC, Pereira CA, Vieira MLFP, Conde WL, Souza PRB (2014). Pesquisa Nacional de Saúde no Brasil: concepção e metodologia de aplicação. Cien Saude Colet.

[29] Stopa SR, Szwarcwald CL, Oliveira MM, Gouvea ECDP, Vieira MLFP, Freitas MPS (2020). Pesquisa Nacional de Saúde 2019: histórico, métodos e perspectivas. Epidemiol Serv Saude.

[30] Claro RM, Monteiro CA (2010). Acurácia da auto-aferição de peso e altura e do índice de massa corporal por meio de entrevista telefônica. Rev Bras Epidemiol.

[31] da Silva LES, Gouvêa ECDP, Stopa SR, Tierling VL, Sardinha LMV, Macario EM (2021). Data Resource Profile: Surveillance System of Risk and Protective Factors for Chronic Diseases by Telephone Survey for adults in Brazil (Vigitel). Int J Epidemiol.

[32] Santos RO, Gubert MB, Vasconcelos FAG (2020). Nutritional status of adolescents and associated factors: a systematic review. Cien Saude Colet.

[33] Sousa TM, Silva LESD, Rodrigues LC, Caldeira TCM, Cardoso LO, Claro RM (2025). Time trend of Vigitel Brasil operation indicators (2006 to 2023). Rev Saude Publica.

[34] World Health Organization (WHO) (2008). Framework and standards for country health information systems.

[35] Levy D, de Almeida LM, Szklo A (2012). The Brazil SimSmoke policy simulation model: the effect of strong tobacco control policies on smoking prevalence and smoking-attributable deaths in a middle income nation. PLoS Med.

[36] Hallal PC, Andersen LB, Bull FC, Guthold R, Haskell W, Ekelund U, Lancet Physical Activity Series Working Group (2012). Global physical activity levels: surveillance, progress, pitfalls, and prospects. Lancet.

[37] Malta DC, Gomes CS, Felisbino-Mendes MS, Veloso GA, Machado IE, Cardoso LO (2024). Undernutrition, and overweight and obesity: the two faces of malnutrition in Brazil, analysis of the Global Burden of Disease, 1990 to 2019. Public Health.

[38] Santos SMC, Ramos FP, Medeiros MAT, Mata MM, Vasconcelos FAG (2021). Avanços e desafios nos 20 anos da Política Nacional de Alimentação e Nutrição. Cad Saude Publica.

[39] Brasil. Ministério da Saúde (2014). Guia alimentar para a população brasileira.

[40] Szwarcwald CL, Leal MC, Esteves-Pereira AP, de Almeida WS, Frias PG, Damacena GN (2019). Avaliação das informações do Sistema de Informações sobre Nascidos Vivos (SINASC), Brasil. Cad Saúde Pública.

[41] Oliveira MM, Andrade SSCA, Dimech GS, de Oliveira JCG, Malta DC, Rabello DL (2015). Avaliação do Sistema de Informações sobre Nascidos Vivos. Brasil, 2006 a 2010. Epidemiol Serv Saude.

[42] World Health Organization (2013). Global and regional estimates of violence against women: prevalence and health effects of intimate partner violence and non-partner sexual violence.

[43] Instituto de Pesquisa Econômica Aplicada (IPEA) ODS 5: Igualdade de gênero [Internet].

[44] Celeste RK, Nadanovsky P, Ponce de Leon A (2011). The role of socioeconomic position in dental caries in adolescents. Rev Saude Publica.

[45] Hebert JR, Clemow L, Pbert L, Ockene IS, Ockene JK (1995). Social desirability bias in dietary self-report may compromise the validity of dietary intake measures. Int J Epidemiol.

[46] Vargas AMD, Teixeira DSC, Alves MCGP, Alencar GP, Bernal RTI, Vasconcelos M (2025). Methodological aspects of national surveys in Brazil: contributions to the debate on oral health surveillance. Braz Oral Res.

[47] Trevilato GC, Riquinho DL, Mesquita MO, Rosset I, Augusto LGS, Nunes LN (2022). Anomalias congênitas na perspectiva dos determinantes sociais da saúde. Cad Saude Pública.

[48] Luquetti DV, Koifman RJ (2010). Qualidade da notificação de anomalias congênitas pelo Sistema de Informações sobre Nascidos Vivos (SINASC): estudo comparativo nos anos 2004 e 2007. Cad Saude Publica.

[49] Oliveira MM, Campos MO, de Andreazzi MAR, Malta DC (2017). Características da Pesquisa Nacional de Saúde do Escolar - PeNSE. Epidemiol Serv Saude.

