# Supplementary material for: Risk and Protective Factor Indicators Management Committee: methodological approach in the construction of indicators of the interagency health information network (Ripsa), 2023
Source: Rev Bras Epidemiol. 2026 Jul 27;29:e260026. doi: 10.1590/1980-549720260026 (PMC13405555; doi:10.1590/1980-549720260026)
Supplement: Translation

# Translation: Comitê de Gestão de Indicadores de Fatores de Risco e Proteção: metodologia utilizada na construção dos indicadores da rede interagencial de informações para a saúde, 2023

**DOI:** 10.1590/1980-549720260026.2

**Authors:** Deborah Carvalho Malta, Max Moura de Oliveira, Mariana Gonçalves de Freitas, Anna Beatriz Souza Antunes, Letícia Mendes Ricardo, Vera Lucia Tierling, Debora França dos Santos, Paula Carvalho de Freitas, Flora Vitória Serena Oliveira Baldi, Bárbara Aguiar Carrato, Ana Lorena Lima Ferreira, Thanise Sabrina Souza Santos, Gabriela Oliveira, Isabela da Costa Gaspar da Silva, Luiz Antônio Alves de Menezes-Júnior, Luci Fabiane Scheffer Moraes, Comitê de Gestão de Indicadores de Fatores de Risco e Proteção

**Affiliations:** I Universidade Federal de Minas Gerais, Escola de Enfermagem, Departamento de Enfermagem Materno-Infantil e Saúde Pública – Belo Horizonte (MG), Brasil.; II Universidade Federal de Goiás, Instituto de Patologia Tropical e Saúde Pública, Departamento de Saúde Coletiva – Goiás (GO), Brasil.; III Ministério da Saúde, Secretaria de Vigilância em Saúde e Ambiente – Brasília (DF), Brasil.; IV Universidade Federal de Minas Gerais, Escola de Enfermagem – Belo Horizonte (MG), Brasil.; V Ministério da Saúde, Secretaria de Atenção Primária à Saúde – Brasília (DF), Brasil.; VI Ministério da Saúde, Secretaria de Informação e Saúde Digital – Brasília (DF), Brasil.

**Keywords:** Indicadores básicos de saúde, Fatores de risco, Fatores de proteção, Inquéritos epidemiológicos, Sistemas de Informação em Saúde, Brasil

## Abstract

**Objetivo:**

Descrever a atuação do Comitê de Gestão de Indicadores de Fatores de Risco e Proteção (CGI FR&P) na retomada e atualização da matriz de indicadores da Rede Interagencial de Informações para a Saúde (Ripsa) em 2023, destacando a sustentabilidade das fontes de dados e dos processos de validação.

**Métodos e Resultados:**

Foram realizadas reuniões técnicas interinstitucionais entre 2023 e 2025, envolvendo 36 instituições, para revisão, definição e validação dos indicadores, com utilização de ficha de qualificação de indicadores padronizada. Foi consensuado o uso dos dados provenientes do Sistema de Vigilância de Fatores de Risco e Proteção para Doenças Crônicas por Inquérito Telefônico (Vigitel), Estudo Nacional de Alimentação e Nutrição (Enani), Sistema de Informações sobre Nascidos Vivos (Sinasc), da Pesquisa Nacional de Saúde (PNS), Pesquisa de Orçamentos Familiares (POF), Pesquisa Nacional de Saúde do Escolar (PeNSE), Pesquisa Nacional de Demografia e Saúde (PNDS), Pesquisa Industrial Anual Produto (PIA-Produto), Pesquisa Nacional de Saúde e Nutrição (PNSN) e Pesquisa Nacional de Saúde Bucal (SB Brasil). A matriz foi ampliada para 39 indicadores, distribuídos em 9 dimensões: doenças crônicas não transmissíveis, consumo de álcool, tabagismo, atividade física, estado nutricional, consumo alimentar, parto, violência e saúde bucal.

**Conclusão:**

O CGI FR&P contribuiu para a qualificação e sustentabilidade dos indicadores da Ripsa, essenciais para o monitoramento de políticas públicas, decisões baseadas em evidências e enfrentamento das desigualdades em saúde, alinhando-se às agendas nacionais e internacionais, como os Objetivos de Desenvolvimento Sustentável (ODS).

## INTRODUÇÃO

A Rede Interagencial de Informações para a Saúde (Ripsa) é uma rede colaborativa criada em 1996 para produção, validação e disseminação de informações estratégicas para a saúde pública no Brasil. Instituída por meio de uma cooperação entre o Ministério da Saúde (MS) e a Organização Pan-Americana da Saúde (OPAS/OMS), o Conselho Nacional de Secretários de Saúde (Conass), o Conselho Nacional de Secretarias Municipais de Saúde (Conasems), em parceria com diversas instituições de ensino, pesquisa e órgãos governamentais, a Ripsa consolidou-se como um fórum fundamental para a geração de informações aplicadas à formulação, gestão e avaliação de políticas e ações do Sistema Único de Saúde (SUS)^1^.

Após quase duas décadas de operação contínua, a Rede entrou em um período de inatividade a partir de 2015. Esse hiato se estendeu até 2023, quando foi retomada, no âmbito da Secretaria de Informação e Saúde Digital (Seidigi/MS), atribuindo-se à secretaria a competência de apoiar seu desenvolvimento e coordenação. A retomada oficial se deu em agosto de 2023, reafirmando o papel da Ripsa como instância técnica qualificadora de informação em saúde^2^.

A Ripsa atualmente é integrada por 45 instituições governamentais e não governamentais, com a participação voluntária de centenas de profissionais especializados, assegurando rigor metodológico e legitimidade técnica na pactuação e a atualização periódica de um conjunto de 184 Indicadores Básicos de Saúde (IDB). Os Comitês de Gestão de Indicadores (CGI) constituem instâncias permanentes responsáveis por elaborar e revisar periodicamente os indicadores, os quais se encontram organizados em sete categorias: demográficos, socioeconômicos, morbidade, mortalidade, recursos, cobertura, e fatores de risco e proteção. Cada tema é coordenado por um comitê técnico, responsável por definir, produzir e analisar os indicadores de saúde^3^.

As instituições atuam em consenso na produção, análise e disseminação de dados, informações e indicadores relativos às condições de saúde e seus determinantes, promovendo o conhecimento da situação sanitária brasileira e subsidiando o monitoramento e a tomada de decisões no âmbito do SUS^1^. O CGI de Fatores de Risco e Proteção (FR&P) foi criado em 2011, na 21^a^ Oficina de Trabalho Interagencial (OTI), instância máxima para deliberação das definições técnicas da Ripsa. A criação deste CGI foi fundamental para ampliar o monitoramento dos indicadores de FR&P^4^. Em sua origem, os indicadores da Ripsa foram organizados conforme a classificação do folheto *Situación de Salud en las Américas: Indicadores Básicos 1995*, incluindo os grupos de mortalidade, morbidade e fatores de risco^5^. Posteriormente, houve a separação dos indicadores de morbidade e fatores de risco. Em agosto de 2023, o CGI FR&P foi recriado, com a participação de 36 instituições de ensino e pesquisa, sob coordenação da Universidade Federal de Minas Gerais (UFMG) e relatoria do Departamento de Análise Epidemiológica e Vigilância de Doenças Não Transmissíveis (Daent) da Secretaria de Vigilância em Saúde e Ambiente (SVSA) do Ministério da Saúde^6^.

Diante desse contexto, o objetivo foi descrever a atuação do Comitê de Gestão de Indicadores de Fatores de Risco e Proteção na retomada da Ripsa em 2023, bem como apresentar suas contribuições e a composição dos indicadores sob sua responsabilidade. O artigo também detalha os processos metodológicos de pactuação, validação e sustentabilidade das fontes de dados que fundamentam a matriz de indicadores de fatores de risco e proteção da Ripsa.

## MÉTODOS E RESULTADOS

Foram realizadas seis reuniões do CGI FR&P entre 2023 e 2025, envolvendo representantes de 36 instituições governamentais e não governamentais, com o objetivo de revisar, selecionar e validar os indicadores, definir fontes de dados e métodos de cálculo, testar e analisar a viabilidade de monitoramento, preparar ficha de qualificação de indicadores e alcançar consensos sobre a matriz de indicadores. Durante essas reuniões, cada indicador foi criteriosamente avaliado quanto à relevância, sustentabilidade e viabilidade, considerando especificidades metodológicas e operacionais.

Foi adotada a ficha de qualificação de indicadores padronizada da Ripsa, que contempla campos obrigatórios como denominação, conceituação, justificativa, método de cálculo, categorias de análise, periodicidade e fonte dos dados, assegurando transparência, padronização e reprodutibilidade das informações. Esse instrumento foi desenvolvido coletivamente de acordo com o consenso entre as instituições participantes, visando garantir comparabilidade e qualidade nas informações produzidas.

No processo de elaboração e revisão dos indicadores, todas as etapas respeitaram os preceitos éticos previstos na legislação brasileira vigente. Foram utilizados exclusivamente dados secundários, de domínio público e desidentificados, sem possibilidade de identificação individual dos participantes, o que dispensou submissão ao sistema de Comitês de Ética em Pesquisa (CEP/Conep), conforme preconiza a Resolução n.° 466, de 12 de dezembro de 2012, do Conselho Nacional de Saúde^7^.

Como critérios para seleção e validação dos indicadores, consideraram-se: relevância para a saúde coletiva, sustentabilidade da fonte de dados, viabilidade operacional, potencial para monitoramento temporal e territorial, e alinhamento com agendas nacionais e internacionais.

Os indicadores do CGI FR&P foram construídos com base em dados extraídos de diferentes inquéritos nacionais e sistemas de informação em saúde, abrangendo distintas populações, metodologias e períodos de coleta. As principais fontes utilizadas foram:

a) Sistema de Vigilância de Fatores de Risco e Proteção para Doenças Crônicas por Inquérito Telefônico (Vigitel): realizado entre os anos de 2006 e 2023. O Vigitel é um inquérito telefônico de base populacional, realizado anualmente pelo Ministério da Saúde, coletando informações sobre as Doenças Crônicas Não Transmissíveis (DCNT) e seus principais FR&P, em adultos de 18 anos e mais, nas capitais brasileiras. Nas edições realizadas entre 2006 e 2019, estabeleceu-se um tamanho amostral mínimo de cerca de 2 mil indivíduos em cada cidade, por meio de telefone fixo. No entanto, nos anos de 2020 e 2021, em virtude de dificuldades impostas pela pandemia de covid-19 à coleta dos dados, estabeleceu-se um tamanho amostral reduzido de cerca de mil indivíduos em cada cidade. Para o ano de 2023, foram realizadas 800 entrevistas em cada uma das localidades e incluídas entrevistas por celular na amostra, devido à queda da cobertura por telefone fixo. São usados pesos de pós-estratificação por sexo, idade e escolaridade para representar os adultos nas capitais^8^.
b) Pesquisa Nacional de Saúde (PNS): constitui a mais ampla pesquisa de saúde do Brasil, realizada em 2013 e 2019. Utiliza subamostra da Amostra Mestra do Sistema Integrado de Pesquisas Domiciliares (SIPD) do Instituto Brasileiro de Geografia e Estatística (IBGE), empregando amostragem conglomerada em três estágios (unidade primária de amostragem, domicílios, indivíduos). O desenvolvimento da pesquisa envolveu a coleta das informações segundo eixos: Condições de saúde; Atenção à saúde (acesso, utilização, continuidade do cuidado e financiamento); Vigilância das DCNT e FR associados, além das questões relacionadas às desigualdades sociais em saúde^9^.
c) Pesquisa de Orçamentos Familiares (POF): realizada pelo IBGE desde 1961, incluindo informações sobre compra de alimentos a partir dos anos 2002/2003, e também sobre consumo alimentar nas edições de 2008/2009 e 2017/2018. A POF investiga os orçamentos familiares combinados com outras informações sobre as condições de vida das famílias brasileiras, os índices de preços, as condições de vida com base no consumo e orçamento das famílias, além de incluir estudos sobre consumo alimentar pessoal e análise da disponibilidade domiciliar de alimentos^10^.
d) Pesquisa Nacional de Saúde do Escolar (PeNSE): realizada em 2009, 2012, 2015 e 2019, constitui a mais importante pesquisa entre adolescentes. A partir de 2015, a PeNSE passou a representar a população de escolares de 13 a 17 anos, em subamostra, enquanto em 2019 a amostra de 13 a 17 anos abrangeu cerca de 150 mil escolares brasileiros, de escolas públicas e privadas. A PeNSE tem como objetivos: determinar a prevalência de fatores de risco comportamentais junto à população de adolescentes; acompanhar as tendências dessas prevalências ao longo do tempo e gerar evidências para orientar e avaliar intervenções^11,12^.
e) Pesquisa Nacional de Demografia e Saúde (PNDS): realizada em 1996 e 2006, a PNDS descreve o perfil da população feminina em idade fértil e de menores de cinco anos no Brasil e identifica as mudanças ocorridas na situação da saúde e da nutrição desses dois grupos. A pesquisa foi realizada por meio de entrevistas domiciliares, incluindo mensurações antropométricas (altura, peso e circunferência da cintura), coleta de amostras de sangue e informações sobre o uso de sal iodado. Em 2006, a amostra foi de 15 mil mulheres e cerca de 5 mil crianças menores de 5 anos, como amostragem representativa das 5 macrorregiões brasileiras e do contexto urbano e rural^13^.
f) Estudo Nacional de Alimentação e Nutrição (Enani): é um inquérito populacional de base domiciliar, com uma primeira edição realizada em 2019 em uma amostra probabilística de crianças menores de 5 anos de idade distribuídas em 123 municípios dos 26 estados da Federação e no Distrito Federal, incluindo amostra de 14.558 crianças até 5 anos. O Enani incluiu indicadores de nutrição e saúde infantil, como amamentação e estado nutricional^14^.
g) Pesquisa Industrial Anual Produto (PIA-Produto): insere-se no Programa de Modernização das Estatísticas Econômicas do IBGE, com o objetivo de atender à crescente demanda por informações estatísticas sobre bens e serviços industriais produzidos no país. Com a série iniciada em 1998, a PIA-Produto tem por objetivo disponibilizar informações atualizadas sobre a produção de bens e serviços industriais, em quantidade e valor, dos produtos fabricados nas principais empresas do país. Os dados analisados referem-se a produção, importação e consumo de álcool no país^15^.
h) Pesquisa Nacional de Saúde e Nutrição (PNSN): realizada em 1989, incluindo uma amostra domiciliar com representatividade nacional. A PNSN possibilitou a coleta sobre amamentação, estado nutricional, características dos domicílios, entre outras informações, contribuindo para o diagnóstico alimentar e nutricional e para o planejamento das políticas públicas^16‘18^.
i) Pesquisa Nacional de Saúde Bucal (SB Brasil): inquérito epidemiológico nacional de saúde bucal, promovido pelo Ministério da Saúde. Realizado com amostragem probabilística em conglomerados, o SB Brasil é representativo para grandes regiões, unidades da federação e capitais, abrangendo diferentes faixas etárias. Permite comparabilidade para as edições em 2010 e 2023. Com base em entrevistas e exames clínicos, foram avaliados indicadores como cárie dentária, consequências clínicas de cárie não tratada (PUFA), necessidades de prótese, entre outros^19^.
j) Sistema de Informações sobre Nascidos Vivos (Sinasc): as informações, por meio da Declaração de Nascido Vivo, referem-se a tipo de parto, gestação, características da mãe e bebê, entre outras. Tais informações são fundamentais, pois possibilitam a construção de indicadores como a taxa de mortalidade infantil, mortalidade neonatal precoce e tardia, frequências relacionadas ao risco de recém-nascidos, número de consultas pré-natal e taxa de fecundidade por local de ocorrência. Com essas informações é possível estimar demandas específicas à organização dos serviços, como a necessidade de atenção intensiva neonatal^16^. O Sinasc foi implantado oficialmente a partir de 1990 e reúne dados sobre os nascimentos informados em todo o território nacional, fornecendo informações sobre natalidade para todos os níveis do SUS^17^.

A matriz de indicadores do CGI FR&P foi ampliada e reestruturada ao longo dos anos. Até 2011, era composta por 17 indicadores. Na revisão mais recente, foram propostos 8 novos indicadores e, conforme deliberação da 31^a^ OTI, cada fonte distinta passou a ser considerada como novo indicador. Em 2025, a matriz conta com 39 indicadores de fatores de risco e proteção, organizados em 9 dimensões temáticas. A Figura 1 apresenta as conexões entre essas dimensões e as fontes de dados dos indicadores, destacando que o tamanho do nó de cada fonte é diretamente proporcional à quantidade de indicadores que utiliza essa fonte como referência. Em síntese, quanto maior o número de indicadores associados a uma determinada fonte de dados, maior será o seu nó na representação gráfica. Na figura, observa-se a relevância da PNS e do Vigitel como fontes primárias para diversos indicadores, sendo 11 e 10 indicadores, respectivamente. A análise de rede evidencia o papel central dessas duas fontes na sustentabilidade do sistema de monitoramento e na distribuição equilibrada dos indicadores entre as nove dimensões.

**Figura 1:** Rede de conexões entre nove dimensões temáticas e fontes de dados dos indicadores de fatores de risco e proteção, Ripsa, Brasil, 2025.

Detalhes referentes ao código, nome, conceito, às fontes de dados e variáveis de análise de cada indicador estão apresentados no Quadro 1 e, mais profundamente, no Material Suplementar 1. As fichas de qualificação completas estão disponíveis no site da Ripsa^20^. As desagregações dos indicadores variam conforme as fontes de dados, mas em geral incluem: unidade geográfica (regiões, estados, Distrito Federal, capitais), idade, sexo e raça/cor – e, quando disponível, também incluem escolaridade e renda, utilizadas como proxies de condição socioeconômica.

**Quadro 1:** Matriz dos indicadores de fatores de risco e proteção nas nove dimensões temáticas, Ripsa, Brasil, 2025.

Código e nome	Conceito	Fontes dados	Categorias de análise
FRP.1.01.1: Prevalência de diabetes mellitus autorreferida em indivíduos de 18 anos ou mais de idade.	Prevalência de diabetes mellitus por diagnóstico médico autorreferido em indivíduos com 18 anos ou mais de idade.	Vigitel 2006 a 2023	**Categorias Vigitel“**
FRP.1.01.2: Prevalência de diabetes mellitus autorreferida em indivíduos de 18 anos ou mais de idade.	Prevalência de diabetes mellitus por diagnóstico médico autorreferido em indivíduos com 18 anos ou mais de idade.	PNS 2013, 2019	**Categorias PNS“”**
FRP. 1.02.1: Prevalência de hipertensão arterial autorreferida em indivíduos de 18 anos ou mais de idade.	Prevalência de hipertensão arterial por diagnóstico médico autorreferido em indivíduos com 18 anos ou mais de idade.	Vigitel 2006 a 2023	**Categorias Vigitel“**
FRP.1.02.2: Prevalência de hipertensão arterial autorreferida em indivíduos de 18 anos ou mais de idade.	Prevalência de hipertensão arterial por diagnóstico médico autorreferido em indivíduos com 18 anos ou mais de idade.	PNS 2013, 2019	**Categorias PNS“”**
FRP.2.01.1: Prevalência de indivíduos de 18 anos ou mais de idade que referem consumo episódico pesado de bebidas alcoólicas.	Percentual de indivíduos de 18 anos ou mais de idade que referem consumo episódico pesado de bebidas alcoólicas nos últimos 30 dias.	Vigitel 2006 a 2023	**Categorias Vigitel“**
FRP.2.01.2: Prevalência de indivíduos de 18 anos ou mais de idade que referem consumo episódico pesado de bebidas alcoólicas.	Percentual de indivíduos de 18 anos ou mais de idade que referiram consumo episódico pesado de bebidas alcoólicas nos últimos 30 dias.	PNS 2013, 2019	**Categorias PNS“”**
FRP.2.02.1: Prevalência de indivíduos com 18 anos ou mais de idade dirigindo veículos motorizados após consumo de bebidas alcoólicas.	Percentual de indivíduos de 18 anos ou mais de idade que referiram dirigir veículos motorizados após consumo de qualquer quantidade de bebidas alcoólicas.	Vigitel 2006 a 2023	**Categorias Vigitel“**
FRP.2.02.2: Prevalência de indivíduos com 18 anos ou mais de idade dirigindo veículos motorizados após consumo de bebidas alcoólicas.	Percentual de condutores de automóvel ou motocicleta que dirigiram logo depois de consumir bebida alcoólica nos últimos 12 meses.	PNS 2013, 2019	**Categorias PNS“”**
FRP.2.03: Consumo de álcool em litros de álcool puro per capita (com 15 anos ou mais) por ano.	Consumo de álcool em litros de álcool puro per capita (com 15 anos ou mais) por ano.	PIA-Produto, COMEX STAT e UN Tourism	Não se aplica
FRP.3.01.1: Prevalência de fumantes atuais de 18 anos ou mais de idade.	Prevalência de indivíduos de 18 anos ou mais de idade que referiram fumar.	Vigitel 2006 a 2023	**Categorias Vigitel“**
FRP.3.01.2: Prevalência de fumantes atuais de 18 anos ou mais de idade.	Prevalência de indivíduos de 18 ou mais anos de idade que referiram fumar.	PNS 2013, 2019	**Categorias PNS“”**
FRP.3.02.1: Prevalência de uso atual de DEF# em indivíduos de 18 anos ou mais de idade.	Prevalência de uso atual de DEF# por indivíduos de 18 anos ou mais de idade.	Vigitel 2019 a 2023	**Categorias Vigitel“**
FRP.3.02.2: Prevalência de uso atual de DEF# em indivíduos de 18 anos ou mais de idade.	Prevalência de uso atual de DEF# por indivíduos de 18 anos ou mais de idade.	PNS 2013, 2019	**Categorias PNS“”**
FRP.3.02.3 Prevalência do uso atual de DEF# em escolares de 13 a 17 anos de idade.	Prevalência de escolares de 13 a 17 anos de idade que referiram ter usado DEF# nos últimos 30 dias.	PeNSE	Sexo; Faixa etária: 13 a 15 anos, 16 a 17 anos; Raça/cor: branca, preta e parda
FRP.3.03.1: Prevalência de ex-fumantes de 18 anos ou mais de idade.	Prevalência de indivíduos de 18 anos ou mais de idade ex-fumantes.	Vigitel 2006 a 2023	**Categorias Vigitel“**
FRP.3.03.2: Prevalência de ex-fumantes de 18 anos ou mais de idade.	Prevalência de indivíduos de 18 anos ou mais de idade ex-fumantes.	PNS 2013, 2019	**Categorias PNS“”**
FRP.4.01.1: Prevalência de prática de nível recomendado de atividade física no tempo livre em indivíduos com 18 ou mais anos de idade.	Prevalência de indivíduos de 18 anos ou mais de idade que praticam o nível recomendado de atividade física no tempo livre.	Vigitel 2006 a 2023	**Categorias Vigitel“**
FRP.4.01.2: Prevalência de prática de atividade física no tempo livre em indivíduos de 18 anos ou mais de idade.	Prevalência de prática de atividade física em nível recomendado no tempo livre em indivíduos com 18 anos e mais de idade.	PNS 2013, 2019	**Categorias PNS“”**
FRP.4.02.1: Prevalência de atividade física insuficiente em indivíduos de 18 anos ou mais de idade.	Prevalência de atividade física insuficiente em indivíduos com 18 ou mais anos de idade.	Vigitel	**Categorias Vigitel“**
FRP.4.02.2: Prevalência de atividade física insuficiente em indivíduos de 18 anos ou mais de idade.	Prevalência de indivíduos de 18 ou mais anos de idade que são insuficientemente ativos.	PNS 2013, 2019	**Categorias PNS“”**
FRP.5.01.1: Prevalência de excesso de peso em indivíduos de 18 anos ou mais de idade.	Prevalência de excesso de peso em indivíduos com 18 anos ou mais de idade.	Vigitel 2006 a 2023 (exceto 2022)	**Categorias Vigitel“**
FRP.5.01.2: Prevalência de excesso de peso em indivíduos de 18 anos ou mais de idade.	Prevalência de excesso de peso em indivíduos de 18 anos ou mais de idade.	PNS 2013, 2019	**Categorias PNS“”**
FRP.5.02: Prevalência de excesso de peso em adolescentes.	Não há base para a construção desta ficha de qualificação.		
FRP.5.03: Prevalência de excesso de peso em crianças menores de 5 anos de idade.	Percentual de crianças < 5 anos com excesso de peso.	PNSN 1989, PNDS 1996 e 2006, Enani 2019	Sexo; Indicador Econômico Nacional; Raça/cor da mãe: branca, preta, parda, amarela e indígena
FRP.5.04: Prevalência de déficit ponderal para a idade em crianças menores de 5 anos de idade.	Percentual de crianças < 5 anos com déficit ponderal para a idade. Déficit ponderal = valores abaixo do escore-z menor que -2 ou percentil menor que 3 do índice de peso para idade (P/I), de acordo com as curvas de crescimento da OMS.	PNSN 1989, PNDS 1996 e 2006, Enani 2019	Sexo; Indicador Econômico Nacional; Raça/cor da mãe: branca, preta, parda, amarela e indígena
FRP.5.05: Prevalência de déficit estatural para a idade em crianças menores de 5 anos de idade.	Percentual de crianças residentes < 5 anos que apresentam déficit estatural para a idade. Déficit estatural = valores abaixo do percentil 3 ou do escore-z -2 do índice de estatura para a idade (E/I), de acordo com as curvas de crescimento da OMS.	PNSN 1989, PNDS 1996 e 2006, Enani 2019	Sexo; Indicador Econômico Nacional; Raça/cor da mãe: branca, preta, parda, amarela e indígena
FRP.5.06: Prevalência de dupla carga da má nutrição em crianças menores de 5 anos de idade.	Percentual de crianças < 5 anos com dupla carga de má nutrição, definida pelo IMC para a idade classificado como magreza acentuada/magreza ou sobrepeso/obesidade, de acordo com as curvas de crescimento infantil da OMS.	PNSN 1989, PNDS 1996 e 2006, Enani 2019	Sexo; Indicador Econômico Nacional; Raça/cor da mãe: branca, preta, parda, amarela e indígena
FRP.6.01: Participação relativa diária per capita das calorias de frutas, verduras e legumes no total de calorias dos alimentos adquiridos no domicílio.	Participação relativa diária per capita das calorias provenientes de FVL no total de calorias de alimentos adquiridos no domicílio para consumo pela população residente.	POF 2008-2009 e 2017-2018	Sexo; Quintil de Indicador Econômico Nacional: 1° quintil, 2° quintil, 3° quintil, 4° quintil e 5° quintil; Raça/cor: branca, preta, parda, amarela e indígena
FRP.6.02: Participação relativa de frutas, verduras e legumes no total de calorias determinado pelo consumo alimentar pessoal. ^@^	Participação relativa diária per capita de calorias provenientes de frutas, verduras e legumes no total de calorias do consumo alimentar pessoal.	POF 2008-2009 e 2017-2018	Sexo; Situação do domicílio: rural e urbano; Raça/cor: branca, preta, parda, amarela e indígena; Indicador econômico nacional
FRP.6.03: Participação relativa de alimentos ultraprocessados no total de calorias determinado pelo consumo alimentar pessoal. ^@^	Participação relativa diária per capita de calorias provenientes de alimentos ultraprocessados sobre o total de calorias do consumo alimentar pessoal.	POF 2008-2009 e 2017-2018	Sexo; Situação do domicílio: rural e urbano; Raça/cor: branca, preta, parda, amarela e indígena; Indicador econômico nacional
FRP.6.04: Prevalência de aleitamento materno exclusivo durante os seis primeiros meses de vida.	Percentual de crianças residentes de 0 a 5 meses e 29 dias (0 a 179 dias) que foram alimentadas exclusivamente com leite materno.	PNDS 2006 e Enani 2019	Sexo; Indicador econômico nacional; Raça/cor da mãe: branca, preta, parda, amarela e indígena; Situação do domicílio: urbano e rural.
FRP.6.05: Prevalência de aleitamento materno continuado em crianças menores de 2 anos de idade.	Percentual de crianças residentes < 2 anos que foram alimentadas com leite materno, diretamente do peito ou por expressão, concomitante à alimentação complementar.	PNDS 2006 e Enani 2019	Sexo; Indicador econômico nacional; Raça/cor da mãe: branca, preta, parda, amarela e indígena; Situação do domicílio: urbano e rural.
FRP.7.01: Proporção de nascidos vivos por idade da parturiente.	Apresenta a proporção de nascidos vivos, por idade da parturiente, em relação ao total de nascidos vivos.	Sinasc	Unidade geográfica: Brasil, grandes regiões, estados, Distrito Federal e capitais; Faixa etária de mães ou parturientes até 19, 20-34, 35-39, 40+
FRP.7.02: Proporção de nascidos vivos com baixo peso ao nascer por idade gestacional no momento do parto.	Proporção de nascidos vivos com peso < 2.500 gramas, por idade gestacional no momento do parto.	Sinasc	Tempo de gestação: pré-termo, a termo/pós-termo ou pós-termo.
FRP.7.03: Prevalência de nascidos vivos com anomalias congênitas.	N.° de nascidos vivos com menção a, pelo menos, uma anomalia congênita, em relação ao total de nascidos vivos registrados.	Sinasc	Unidade geográfica: Brasil, grandes regiões, estados, Distrito Federal e capitais.
FRP.8.01: Prevalência de violências contra mulheres com 18 anos ou mais de idade.	Percentual de mulheres ≥ 18 anos que referiram ter sofrido pelo menos algum dos tipos de violência perguntados, nos últimos 12 meses.	PNS 2013, 2019	**Categorias PNS“”**
FRP.8.02: Prevalência de violência sexual contra meninas de 13 a 17 anos de idade.	Prevalência de meninas de 13 a 17 anos de idade que referiram ter sofrido violência sexual alguma vez na vida.	PeNSE	Faixa etária: 13 a 15 anos e 16 a 17 anos; Raça/cor: branca, preta e parda.
FRP.9.01: Índice CPO-D (n° de dentes cariados, perdidos e obturados) aos 12 anos de idade.	Número médio de dentes permanentes cariados, perdidos (extraídos) e obturados (restaurados), aos 12 anos de idade.	SB Brasil	Ano: 1996, 2003, 2010 e 2023.
FRP.9.02: Proporção de crianças de 5 a 6 anos de idade livres de cárie (índice ceo-dl = 0)	Percentual de crianças de 5 a 6 anos de idade com índice ceo-d1 = 0.	SB Brasil	Ano: 1996, 2003, 2010 e 2023.

IMC: Índice de Massa Corporal; FVL: frutas, verduras e legumes; Vigitel: Sistema de Vigilância de Fatores de Risco e Proteção para Doenças Crônicas por Inquérito Telefônico; PNS: Pesquisa Nacional de Saúde; PIA-Produto: Pesquisa Industrial Anual Produto do Instituto Brasileiro de Geografia e Estatística; COMEX STAT: Sistema de Consultas e Extração de Dados do Comércio Exterior Brasileiro do Ministério do Desenvolvimento, Indústria, Comércio e Serviços; UN Tourism: Estatísticas de turismo da Organização Mundial do Turismo da Organização das Nações Unidas; PeNSE: Pesquisa Nacional de Saúde do Escolar; PNSN: Pesquisa Nacional sobre Saúde e Nutrição; PNDS: Pesquisa Nacional de Demografia e Saúde; Enani: Estudo Nacional de Alimentação e Nutrição Infantil; POF: Pesquisa de Orçamentos Familiares; Sinasc: Sistema de Informações sobre Nascidos Vivos; SB Brasil: Pesquisa Nacional de Saúde Bucal; #Dispositivo Eletrônico de Fumar; “Sexo; Faixa etária; Nível de instrução; “”Sexo; Faixa etária; Nível de instrução; Rendimento domiciliar; Raça/cor; Situação do domicílio; Renda); ^@^ - Indicador em Construção.

### Declaração de disponibilidade de dados

Todo o conjunto de dados que dá suporte aos resultados deste estudo foi publicado no artigo e na seção “Materiais suplementares”.

## DISCUSSÃO

O estudo abordou a relevância do processo metodológico conduzido pelo CGI FR&P na retomada da Ripsa, culminando em uma matriz de indicadores alinhada aos desafios contemporâneos da saúde pública brasileira. A reativação da Rede em 2023 ocorre após um hiato de quase uma década, período marcado por profunda instabilidade política e pela implementação de políticas de austeridade fiscal, como a Emenda Constitucional n.° 95, que impuseram severas restrições ao financiamento de um sistema de saúde universal que, apesar de seus avanços históricos, enfrenta desafios crônicos^21,22^.

Nesse contexto adverso, a capacidade de articulação da comunidade de saúde pública se mostrou resiliente. Um exemplo foi a produção do “Caderno especial de indicadores básicos para o monitoramento da covid-19”, uma iniciativa que aplicou o método colaborativo e a expertise da rede para responder à emergência sanitária, mesmo sem a chancela formal da Ripsa^23^. Portanto, a retomada oficial da rede não é apenas uma reativação burocrática, mas uma reafirmação política da importância da governança baseada em evidências para o fortalecimento e a sustentabilidade do SUS.

A abordagem adotada pelo comitê reflete uma notável flexibilidade para alinhar a vigilância nacional às prioridades globais e locais, garantindo a pertinência da informação gerada. A matriz de indicadores dialoga diretamente com as metas pactuadas nos Objetivos de Desenvolvimento Sustentável (ODS)^24,25^, o Plano de Ação Global para Enfrentamento das DCNT da Organização Mundial da Saúde (OMS)^26^ e, fundamentalmente, com o Plano de Ações Estratégicas para o Enfrentamento das Doenças Crônicas Não Transmissíveis no Brasil 2011-2022^26^ e o Plano de Ações Estratégicas para o Enfrentamento das Doenças Crônicas e Agravos Não Transmissíveis no Brasil 2021-2030^26^. Essa capacidade de revisar e adaptar os indicadores é fundamental para que os dados subsidiem efetivamente a Atenção Primária à Saúde (APS), que é a ordenadora do cuidado e o lócus principal para a implementação de ações de prevenção e controle desses agravos. A disponibilização de dados consistentes e regulares fortalece o planejamento territorial e a avaliação de programas na APS e as estratégias de promoção da saúde^26,27^.

O desenho da matriz privilegia fontes de dados de reconhecida qualidade e robustez metodológica, um pilar para a credibilidade dos indicadores. O sistema de inquéritos de saúde do Brasil, com sua complexidade e abrangência, é um ativo estratégico para a vigilância, e a centralidade da PNS e do Vigitel, evidenciada na análise de rede, confirma seu papel complementar e sinérgico. A PNS oferece profundidade e representatividade nacional, sendo insubstituível para análises de desigualdades sociais, raciais e regionais em saúde^28,29^. Por sua vez, o Vigitel, cuja validade e acurácia têm sido consistentemente demonstradas em estudos comparativos^30,31^, garante a oportunidade e a capilaridade para o monitoramento anual de tendências nas capitais, sendo uma ferramenta de vigilância ágil e de menor custo. A sustentabilidade dessas fontes é, portanto, vital. Contudo, persistem desafios importantes, como a ausência de um monitoramento nutricional regular para adolescentes – uma lacuna crítica que dificulta a vigilância de fatores de risco em uma fase decisiva da vida^32^ – e os desafios inerentes à manutenção de altas taxas de participação em inquéritos em um cenário de mudanças nos padrões de comunicação^33^.

A organização da matriz em dimensões temáticas é outra fortaleza metodológica. Essa estrutura segmenta a complexidade dos dados de forma coerente, alinhando-se a boas práticas internacionais de organização de sistemas de informação em saúde, que preconizam arranjos lógicos para facilitar a análise e o uso da informação^34^. Os indicadores de DCNT, tabagismo, álcool, alimentação inadequada e atividade física permitem um monitoramento integrado dos principais fatores de risco modificáveis. O Brasil é considerado um caso de sucesso no controle do tabagismo, resultado de políticas regulatórias robustas^35^, mas enfrenta constantemente o desafio da interferência da indústria do tabaco, que busca, por exemplo, introduzir novos produtos, como os dispositivos eletrônicos para fumar^35^. Em contrapartida, o consumo de álcool permanece elevado e a inatividade física é um problema de saúde pública global^36^, exigindo vigilância contínua para subsidiar políticas de promoção da saúde e de ambientes saudáveis.

As dimensões de Estado Nutricional e Consumo Alimentar refletem a complexa transição nutricional do país, marcada pela coexistência da desnutrição e do excesso de peso, a chamada dupla carga da má nutrição^37^. O monitoramento do consumo de alimentos saudáveis e não saudáveis, por exemplo, é fundamental para avaliar o impacto de um ambiente alimentar obesogênico e guiar políticas regulatórias, como a taxação e a rotulagem de alimentos^38,39^.

A dimensão Parto e Nascimento, baseada no robusto Sinasc, é vital para o acompanhamento da saúde materno-infantil, permitindo avaliar desde a qualidade do pré-natal até desfechos que têm repercussões por todo o ciclo de vida, como o baixo peso ao nascer^40,41^.

Já a inclusão das dimensões de Violência e Saúde Bucal representa um avanço significativo, ampliando o escopo da vigilância para além dos fatores de risco tradicionais. A violência, especialmente contra mulheres, é um grave problema de saúde pública com profundas raízes sociais, e sua mensuração por meio de inquéritos é essencial para dar visibilidade ao fenômeno e subsidiar políticas intersetoriais de proteção^42^. Os indicadores incluídos na matriz estão intimamente relacionados com os ODS 5 (meta 5.2), que visam à eliminação de toda forma de violência contra meninas e mulheres^43^. A saúde bucal, por sua vez, é um marcador sensível de desigualdades sociais, e o monitoramento de indicadores como a prevalência de cárie permite avaliar o acesso a serviços e o impacto de políticas de prevenção^44^.

Essa organização multidimensional não apenas facilita a interpretação dos dados, mas também otimiza a mobilização de experts em áreas específicas, promovendo um aprofundamento técnico em cada campo.

Ao utilizar múltiplas fontes, é imperativo reconhecer e manejar a possibilidade de vieses e de diferenças nas prevalências entre fontes, dados de distintas amostragens e métodos de coleta. Dados de inquéritos populacionais (PNS, Vigitel, PeNSE, Enani, SB Brasil) estão sujeitos a vieses de informação, como o de recordatório ou de desejabilidade social, que podem levar à subestimação de comportamentos de risco, como o baixo consumo de alimentos saudáveis^45^, e à superestimação de práticas saudáveis^28,46^, embora não afetem o monitoramento, dada sua constância ao longo do tempo. Por outro lado, os sistemas de informação, como o Sinasc, embora tenham avançado enormemente em cobertura e qualidade, ainda podem apresentar inconsistências de preenchimento, que exigem processos contínuos de qualificação dos dados^41^. A transparência na documentação desses limites, detalhada nas fichas de qualificação de cada indicador, é um compromisso da Ripsa com o uso criterioso e informado da informação, permitindo que gestores e pesquisadores compreendam as potencialidades e as restrições de cada métrica.

Apesar das limitações inerentes, as fontes escolhidas seguem em constante processo de aprimoramento, refletindo o amadurecimento da vigilância em saúde no Brasil. Um exemplo emblemático é o Sinasc, que tem qualificado progressivamente o registro de anomalias congênitas, ampliando a capacidade do país de monitorar esses eventos e de planejar a atenção à saúde para as crianças afetadas, inclusive sob a ótica dos determinantes sociais^47,48^. Da mesma forma, os inquéritos como a PeNSE evoluem a cada edição para incluir temas emergentes, como saúde mental e bullying, demonstrando a capacidade de adaptação do sistema de vigilância para responder a novos desafios^49^. Esse esforço contínuo de qualificação das fontes reforça a confiabilidade da matriz de indicadores e sua utilidade para a saúde pública.

A reativação da Ripsa e a reorganização do CGI FR&P representam um avanço crucial para a saúde pública brasileira, respondendo diretamente ao objetivo deste artigo de descrever esse processo. A matriz de indicadores, sustentada por fontes de dados de alta qualidade e por um método colaborativo e transparente, reafirma-se como um instrumento estratégico para o monitoramento da saúde da população, para o subsídio de políticas baseadas em evidências e para o fortalecimento do controle social. A garantia de sua sustentabilidade financeira e institucional e o contínuo aprimoramento das fontes de dados são fundamentais para que a informação em saúde cumpra seu papel de orientar a ação e promover o direito universal à saúde no Brasil.

## MATERIAL SUPLEMENTAR

O banco de dados ainda não está disponivel para uso público.

## COMITÊ DE ÉTICA EM PESQUISA

Por se tratar de estudo com dados secundários, de domínio público, anonimizados e sem possibilidade de identificação individual, dispensou-se a submissão ao Comitê de Ética em Pesquisa.
